# Langerhans cell histiocytosis following Hodgkin lymphoma: a case report from Iran

**Published:** 2010

**Authors:** Nahid Reisi Dehkordi, Parvin Rajabi, Azar Naimi, Mitra Heidarpour

**Affiliations:** a Assistant Professor of Pediatrics, Department of Pediatrics, Faculty of Medicine, Isfahan University of Medical Science, Isfahan, Iran; b Professor of Pathology, Department of Pathology, Faculty of Medicine, Isfahan University of Medical Sciences, Isfahan, Iran; c Pathologist, Department of Pathology, Faculty of Medicine, Isfahan University of Medical Sciences, Isfahan, Iran; d Assistant Professor of Pathology, Department of Pathology, Faculty of Medicine, Isfahan University of Medical Sciences, Isfahan, Iran

**Keywords:** Hodgkin Disease, Langerhans Cell Histiocytosis, Immunohistochemistry

## Abstract

The occurrence of Langerhans Cell Histiocytosis (LCH) in a patient with lymphoma is an indication of a probable relationship between them. The two conditions have similarities both clinically and histopathologically. Occurrence of these two conditions in the same patient, particularly not simultaneously, is rare. According to different management and treatment of these conditions, exact histopathologic evaluation and even using immunohistochemistery (IHC) can prevent misdiagnosis. In this report, a 10 year old boy presented who afflicted with LCH 3 years after diagnosis and treatment of mixed cellularity Hodgkin lymphoma.

Langerhans Cell Histiocytosis (LCH) comprises a group of diseases determined with proliferation of Langerhans cells.^1^ The etiology is unknown, although viral causes, reactive immunologic responses and genetic factors have been suggested.^2^ The progressive characteristics of the disease and its response to cancer therapies depict its neoplastic nature.^3^ Its occurrence with other neoplasia, particularly lymphoma, has been reported.^2^ The most common form is simultaneous diagnosis. Occurrence of LCH after Hodgkin lymphoma is seen in less than 0.3% of cases.^4^

So presentation of LCH after Hodgkin Lymphoma is rare and generally has been the subject of isolated case reports.

## Case Report

**Figure 1:**
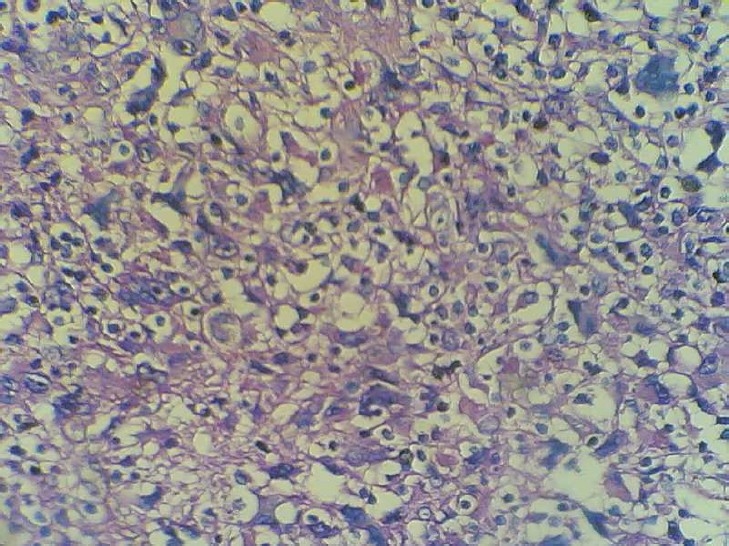
Mixed cellularity type of Hodgkin(HE ×400)

A boy, now 10 years old, presented six years ago (in 2003) with cervical and submandibular lymphadenopathy without hepatosplenomegally and lymph node enlargement in other parts of the body. Concurrent systemic signs (B symptoms) were not found. Complete blood count, erythrocyte sedimentation rate, liver function tests, chest X- ray and bone marrow examination were all normal. Biopsy was performed from one of the enlarged cervical nodes (measuring 2x2x2 cm). In histopathologic examination, in a background of plasma cells, eosinophils and lymphocytes, some Reed- Sternberg cells (classic or mononuclear forms) with prominent large eosinophilic nucleoli were seen (Figure 1).

Diagnosis of Hodgkin lymphoma (mixed cellularity type) was made and the patient was treated with six alternative cycles of ABVD (Adriamycin, Bleomycin, Vinblastine, Dacarbizine) and MOPP (Nitrogen mustard, Vincristine, Prednisolone, Procarbazine) chemotherapy. He responded to this regimen but 1 year later, an asymptomatic mediastinal mass was detected in chest X- ray. Sono- guided biopsy revealed relapse of Hodgkin Lymphoma. The patient underwent treatment with three cycles of chemotherapy with CEP (CCNU, Etoposide, and Prednisolone) and involved field radio-therapy. He responded favorably and was symptom free till 2007.

**Figure 2:**
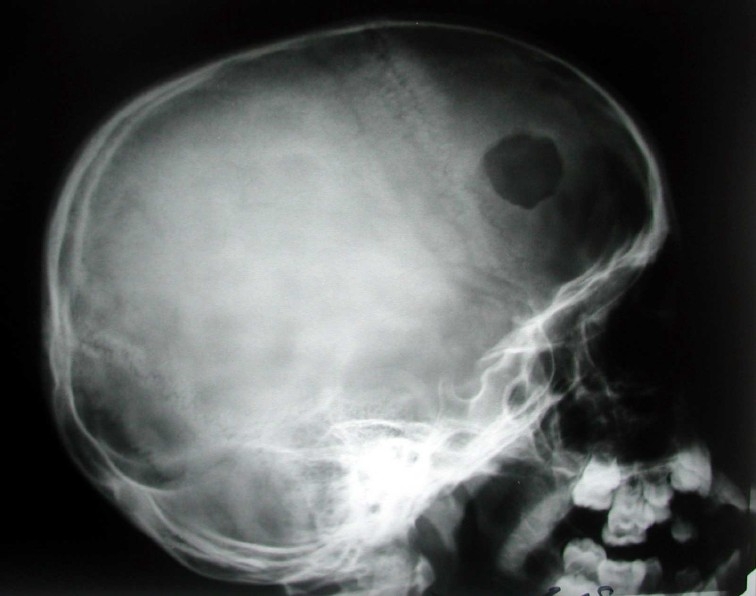
Lytic lesion in the skull

In January 2007, the patient presented with a swelling in his scalp. In skull radiography, an osteolytic lesion in a fairly round shape with the greatest diameter of 3.5 cm was seen on the left side (Figure 2). In bone biopsy, diffuse neoplastic proliferation of Langerhans cells was seen.

These cells depicted acidophilic cytoplasm and lobulated indented nuclei, some with longitudinal clefts. Eosinophils, neutrophils, multinuclear giant cells and foamy macrophages were seen in the background. In addition, some fibrotic bands existed in this background. The histological diagnosis was LCH which was confirmed by IHC staining (positive S100, negative CD15 and CD30). Unfortunately, CD1a marker was not available for IHC study. Previous slides of patient’s lymph node were reexamined, confirming the diagnosis of mixed cellularity Hodgkin Lymphoma. In IHC staining, classic and mononuclear Reed- Sternberg cells reacted with CD15 and CD30 and were negative for S100. CD3 was positive in back-ground lymphocytes while CD20 was negative.

In this way, morphologic and IHC findings confirmed the diagnosis of LCH following Hodgkin Lymphoma.

## Discussion

Overall, the occurrence of lymphoma and LCH in the same individual is not common. More-over, the association of LCH and Hodgkin Lymphoma is rare.^5^,^6^

LCH has a complex relationship with malignant lymphoma. It can occur before, after or simultaneously with Hodgkin Lymphoma.^5^ The exact etiology is unknown. However, in the case of LCH following Hodgkin Lymphoma, reactive proliferation of Langerhans cells in response to radiotherapy and chemotherapy for Hodgkin lymphoma has been considered as a probable cause.^7^

Concurrent occurrence of these diseases and even a report indicating simultaneous nodular sclerosis Hodgkin’s disease, LCH and multiple myeloma without past history of radiotherapy and chemotherapy can make the occurrence of LCH following radiotherapy and chemotherapy questionable.^8^

The time interval between LCH occurrence and previous lymphoma is variable. Intervals of 1 to 33 years have been reported in the literature.^6^ In the case presented here, this interval was about 4 years.

This condition should be considered in the differential diagnosis of recurrent lymphoma. Differentiation of the two diseases is very crucial because of their far different management. Immunohistochemistry can be helpful in such circumstances.

In concurrent occurrence of these diseases, Langerhans cells are smaller than regular ones in histopathologic examination. Abnormal neoplastic proliferation of stromal cells is a response to Hodgkin lymphoma microenvironment. Smaller size of these cells makes differential diagnosis more difficult.^2^

It is interesting that differentiation of these diseases, particularly in the simultaneous from, is considered a pitfall even in PET (Positron Emission Tomography) imaging because both of them show increased uptake in this technique.^9^ In such cases, IHC can confirm the diagnosis of LCH. Therefore, considering the probability of occurrence of these two conditions in the same individual and using appropriate methods to differentiate them are important to avoid misdiagnosis.

## Conclusions

Although rare, the probable occurrence LCH following Hodgkin lymphoma should be kept in mind. Exact evaluation of histopathologic slides and IHC are helpful in making the correct diagnosis.

## References

[1] Howarth DM, Gilchrist GS, Mullan BP, Wiseman GA, Edmonson JH, Schomberg PJ (1999). Langerhans cell histiocytosis: diagnosis, natural history, management, and outcome. Cancer.

[2] Adu- Poku K, Thomas DW, Khan MK, Holgate CS, Smith MEF (2005). Langerhans cell histiocytosis in sequential discordant lymphoma. J Clin Pathol.

[3] Willman CL (1994). Detection of clonal histiocytes in Langerhans cell histiocytosis: biology and clinical significance. Br J Cancer.

[4] Burns BF, Colby TV, Dorfman RF (1983). Langerhans’ cell granulomatosis (histiocytosis X) associated with malignant lymphomas. Am J Surg Pathol.

[5] Egeler RM, Neglia JP, Puccetti DM, Brennan CA, Nesbit ME (1993). Association of Langerhans cell histiocytosis with malignant neoplasms. Cancer.

[6] Egeler RM, Neglia JP, Aricò M, Favara BE, Heitger A, Nesbit ME (1998). The relation of Langerhans cell histiocytosis to acute leukemia, lymphomas, and other solid tumors. Hematol Oncol Clin North Am.

[7] Shin MS, Buchalter SE, Ho KJ (1994). Langerhans cell histiocytosis associated with Hodgkin’s disease: a case report. J Natl Med Assoc.

[8] Ibarrola de Andrés C, Toscano R, Lahuerta JJ, Martínez- González MA (1999). Simultaneous occurrence of Hodgkin’s disease, nodal Langerhans’ cell histiocytosis and multiple myeloma IgA (kappa). Virchows Arch.

[9] Naumann R, Beuthien- Baumann B, Fischer R, Kittner T, Bredow J, Kropp J (2002). Simultaneous occurrence of Hodgkin’s lymphoma and eosinophilic granuloma: a potential pitfall in positron emission tomography imaging. Clin Lymphoma.

